# Farrerol Directly Targets GSK-3*β* to Activate Nrf2-ARE Pathway and Protect EA.hy926 Cells against Oxidative Stress-Induced Injuries

**DOI:** 10.1155/2020/5967434

**Published:** 2020-01-28

**Authors:** Chaoqun Yan, Xiaoyan Zhang, Junqiu Miao, Hongxia Yuan, Enli Liu, Taigang Liang, Qingshan Li

**Affiliations:** ^1^School of Pharmaceutical Science, Shanxi Medical University, Taiyuan 030001, China; ^2^Shanxi Key Laboratory of Chronic Inflammatory Targeted Drugs, School of Traditional Chinese Materia Medical, Shanxi University of Chinese Medicine, Taiyuan 030619, China

## Abstract

Oxidative stress-mediated endothelial injury is considered to be involved in the pathogenesis of various cardiovascular diseases. Farrerol, a typical natural flavanone from the medicinal plant *Rhododendron dauricum* L., has been reported to show protective effects against oxidative stress-induced endothelial injuries in our previous study. However, its action molecular mechanisms and targets are still unclear. In the present study, we determined whether farrerol can interact with glycogen synthase kinase 3*β*- (GSK-3*β*-) nuclear factor erythroid 2-related factor 2- (Nrf2-) antioxidant response element (ARE) signaling, which is critical in defense against oxidative stress. Our results demonstrated that farrerol could specifically target Nrf2 negative regulator GSK-3*β* and inhibit its kinase activity. Mechanistic studies proved that farrerol could induce an inhibitory phosphorylation of GSK-3*β* at Ser9 without affecting the expression level of total GSK-3*β* protein and promote the nuclear translocation of Nrf2 as well as the mRNA and protein expression of its downstream target genes heme oxygenase-1 (HO-1) and NAD(P)H: quinone oxidoreductase 1 (NQO1) in EA.hy926 cells. Further studies performed with GSK-3*β* siRNA and specific inhibitor lithium chloride (LiCl) confirmed that GSK-3*β* inhibition was involved in farrerol-mediated endothelial protection and Nrf2 signaling activation. Moreover, molecular docking and molecular dynamics studies revealed that farrerol could bind to the ATP pocket of GSK-3*β*, which is consistent with the ATP-competitive kinetic behavior. Collectively, our results firstly demonstrate that farrerol could attenuate endothelial oxidative stress by specifically targeting GSK-3*β* and further activating the Nrf2-ARE signaling pathway.

## 1. Introduction

Cardiovascular diseases are widely recognized as the major cause of disability and mortality [1]. Extensive literature suggests that endothelial dysfunction is the initial step in the pathogenesis of atherosclerosis and associated with most forms of cardiovascular disease, such as hypertension, coronary artery disease, chronic heart failure, peripheral artery disease, diabetes, and chronic renal failure [2, 3]. Growing evidence reveals oxidative stress accounts for a significant proportion of the endothelial function impairment in cardiovascular diseases [4]. Excessive production of reactive oxygen species (ROS) disturbs the balance between oxidant and antioxidant intracellular systems and leads to endothelial cell dysfunction and ultimately apoptosis or necrosis. Therefore, it is thought that protecting endothelial cells against oxidative stress could be beneficial for preventing cardiovascular diseases and could aid in the treatment.

In the past decades, epidemiological and mechanistic studies have indicated that dietary intake of flavonoids is associated with a reduced risk for cardiovascular disease and the biological activities of flavonoids are related to their antioxidative effects [5–7]. As the major flavonoids found in citrus fruits and juices, flavanones contribute substantially to the total daily flavonoid intake and are responsible for cardiovascular disease prevention [8]. The prospective study conducted by Cassidy et al. demonstrated flavanone intake was inversely associated with the risk of ischemic stroke [9]. Besides, Mink et al. suggested that flavanone consumption was conversely related to coronary heart disease-induced mortality [10]. Moreover, two main citrus flavanones naringenin and hesperidin were reported to improve endothelial dysfunction, and eriodictyol, another citrus flavanone, was found to protect endothelial cells against oxidative stress-induced cell death [11–13].

**Figure 1:**
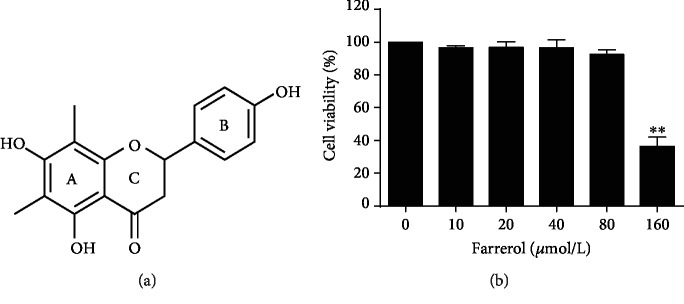
Effects of farrerol on the cell viability of EA.hy926 cells. (a) Chemical structure of farrerol. (b) The cytotoxic effects of farrerol on EA.hy926 cells were determined at various concentrations for 24 h using a MTT assay. Data are presented as means ± SD (*n* = 5). ^∗∗^*p* < 0.01 versus control group.

Farrerol is the major bioactive component isolated from the leaves of traditional Chinese herb *Rhododendron dauricum* L. As a naturally occurring flavanone, it (Figure 1(a)) has the same core structure as naringenin but contains two additional methyl groups on the ring A. It has been documented that farrerol possesses many biological activities, such as antioxidative, anti-inflammatory, antibacterial, anticancer, and inhibition of VSMC proliferation [14–18]. Recent studies from our group also indicate that farrerol has a positive influence on cardiovascular disease. In specific, *in vitro* studies showed that farrerol exerts protective effects against hydrogen peroxide- (H_2_O_2_-) induced apoptosis and endothelial tight junction disruption in human endothelium-derived EA.hy926 cells [19, 20]. Further *in vivo* studies revealed that farrerol reduces the systolic blood pressure and attenuates the aortic lesion in spontaneously hypertensive rats [21].

The positive influence of flavonoids on various cardiovascular diseases is not only attributed to their direct antioxidant properties, such as direct ROS scavenging activity and transient metal chelation, but also their ability of induction endogenous antioxidant systems [6]. Nuclear factor erythroid 2-related factor 2 (Nrf2) is an important regulator of endogenous antioxidant systems. As a key transcription factor, Nrf2 regulates the basal and inducible expression of a battery of antioxidant genes and other cytoprotective phase II detoxifying enzymes and therefore is critical for the maintenance of cellular redox homeostasis. Under normal conditions, Nrf2 is sequestered in the cytoplasm where it forms an inactive complex with the ubiquitin E3 ligase adapter Kelch-like epichlorohydrin-associated protein 1 (Keap1) and rapidly degraded by ubiquitin proteasome, while under oxidative stress conditions, Nrf2 dissociates from Keap1 and translocates into the nucleus to bind to antioxidant response element (ARE) sequence in the promoter region of antioxidant enzyme genes and initiate the gene expression [22]. The evidence from recent studies suggests that glycogen synthase kinase 3*β* (GSK-3*β*), a ubiquitously expressed serine/threonine protein kinase, is a Keap1-independent Nrf2 regulator, which was originally recognized as a negative regulator of glycogen synthesis. The DSGIS motif in the Neh6 domain of Nrf2 is phosphorylated by GSK-3*β*, a modification that facilitates the S-phase kinase-associated protein-1- (SKP1-) CUL1-F box protein- (SCF-) mediated ubiquitylation and proteasomal degradation of Nrf2 [23].

It is previously reported that farrerol attenuates oxidative stress through the Keap1/Nrf2 pathway in BV-2 cells and RAW 264.7 cells [14, 24]. However, the precise role of GSK-3*β* in the antioxidant effects of farrerol in endothelial cells remains to be elucidated. The aim of this study was therefore to explore the possible role of GSK-3*β* in farrerol-mediated protective effects against H_2_O_2_-induced oxidative stress in human endothelium-derived EA.hy926 cells.

## 2. Materials and Methods

### 2.1. Materials

Farrerol (National Institute for the Control of Pharmaceutical and Biological Products, Beijing, China; analytical grade, 99% purity) was dissolved in dimethylsulfoxide (DMSO), stored at -20°C, and then diluted to test concentrations with culture medium or assay buffer immediately prior to the experiment. In all experiments of this study, the final concentration of DMSO was inferior to 0.5% (*v*/*v*) and the control group contained an equal amount of DMSO. Fetal bovine serum (FBS) was purchased from CellMax (Lanzhou, China). Dulbecco's modified Eagle's medium (DMEM) was obtained from Thermo Fisher Scientific (Beijing, China). Antibodies against GSK-3*β* and phospho-GSK-3*β* (Ser9) were purchased from Cell Signaling Technology (Danvers, MA, USA). Antibodies for Nrf2, heme oxygenase-1 (HO-1), NAD(P)H: quinone oxidoreductase 1 (NQO1), Akt, and phospho-Akt were purchased from Abcam (Cambridge, UK). The antibody against *β*-actin and horseradish peroxidase- (HRP-) conjugated secondary antibodies were obtained from ZSGB-BIO (Beijing, China). An antibody for glyceraldehyde 3-phosphate dehydrogenase (GAPDH) was purchased from Bioworld Technology (MN, USA). The antibody against Lamin B1, fluorochrome-conjugated anti-rabbit secondary antibody (Alexa Fluor 555), and the nuclear and cytoplasmic protein extraction kit were obtained from Beyotime Biotechnology Co. (Shanghai, China). 4′,6-Diamidino-2-phenylindole (DAPI), bovine serum albumin (BSA), RIPA lysis buffer, 5x SDS-PAGE sample loading buffer, BCA protein assay kit, and enhanced chemiluminescence (ECL) detection kit were purchased from BOSTER Biological Technology (Wuhan, China). RNAiso Plus, PrimeScript™ RT reagent kit, and SYBR Premix Ex Taq™ II were obtained from Takara Bio, Inc. (Shiga, Japan). M-PER mammalian protein extraction reagent was purchased from Pierce (Pierce, Rockford, USA). Complete protease inhibitor cocktail tablets and pronase were obtained from Roche Applied Science (Mannheim, Germany). Human recombinant GSK-3*β* and phospho-glycogen synthase peptide-2 were purchased from Millipore (Dundee, UK). Kinase-Glo Luminescent Kinase Assay and ADP-Glo™ Kinase Assay were obtained from Promega Corporation (Madison, WI, USA). Triton-X 100, 4% paraformaldehyde, and 3-(4,5-dimethylthiazol-2-yl)-2,5-diphenyltetrazolium bromide (MTT) were purchased from Solarbio Science & Technology Co., Ltd. (Beijing, China). The transfection reagent siRNA-Mate™ was obtained from Genepharm Co., Ltd. (Shanghai, China). GSK-3*β* inhibitor SB415286, adenosine 5′-triphosphate disodium salt hydrate (ATP), and lithium chloride (LiCl) were purchased from Sigma-Aldrich (St. Louis, MO, USA). All other chemicals and reagents were of analytical grade from China, unless otherwise noted.

### 2.2. Cell Culture

Human endothelium-derived EA.hy926 cells were obtained from Cell Bank of Type Culture Collection of the Chinese Academy of Sciences (Shanghai, China) and used within 12 passages for all the experiments. The cells were cultured in high-glucose DMEM supplemented with 10% FBS, penicillin (100 units/mL), and streptomycin (100 *μ*g/mL) at 37°C in a humidified atmosphere containing 5% CO_2_.

### 2.3. Measurement of Cell Viability

MTT quantitative colorimetric assay was performed to estimate cell viability. Briefly, EA.hy926 cells were seeded at a density of 2 × 10^4^ cells per well in 96-well plates and cultured overnight. Then, the cells were subjected to different treatments. Subsequently, 10 *μ*L of MTT stock solution (5 mg/mL) was added to each well. After incubation at 37°C for 4 h, the resultant formazan crystals were dissolved in 100 *μ*L of DMSO and the absorbance values at 490 nm were measured using a SpectraMax i3x microplate reader. Percentage viability was calculated using the following formula: cell viability (%) = OD_Test_/OD_Control_ × 100%.

### 2.4. Protein Extraction

EA.hy926 cells (1 × 10^5^ cells/well) were seeded in 6-well plates. After attachment, cells were treated for 1 h, 3 h, 6 h, 12 h, and 24 h with various concentrations of farrerol (0, 10, 20, 40, and 80 *μ*mol/L). After each experiment, the cells were washed twice with cold phosphate-buffered saline (PBS) and then scraped from the plate with RIPA buffer containing protease and phosphatase inhibitors. After 60 min on ice, the whole cell lysate was centrifuged at 13000 rpm for 15 min at 4°C. Nuclear and cytoplasmic fractions of cell were prepared using the nuclear and cytoplasmic protein extraction kit according to the manufacturer's instruction. The protein concentration in the supernatant was determined using the BCA protein assay, and all the samples in the same experiment were normalized to the equal protein concentration.

### 2.5. Western Blot Analysis

Sample proteins (40 *μ*g) were separated by sodium dodecyl sulfate-polyacrylamide gel electrophoresis (SDS-PAGE) and transferred to nitrocellulose membranes. After the membranes were blocked with 5% skim milk, they were incubated overnight at 4°C with the indicated antibodies. Then, the membranes were washed three times for 10 min each with Tris-buffered saline containing 0.05% Tween 20 (TBST) and incubated for 1 h at room temperature with horseradish peroxidase-conjugated secondary antibody. Proteins in the membranes were visualized by ECL detection kits. Finally, the protein band intensities were quantified using ImageJ software (NIH, Bethesda, MD, USA) and normalized by the *β*-actin or Lamin B1 band intensity.

### 2.6. Quantitative Real-Time Polymerase Chain Reaction (qRT-PCR)

The total RNA of EA.hy926 cells treated with farrerol at various concentrations (0, 10, 20, and 40 *μ*mol/L) were extracted using RNAiso Plus Kit. The RNA content and purity were determined by measuring the optical density at 260 nm and 280 nm. Then, template cDNA for real-time PCR was synthesized using PrimeScript™ RT reagent kit. The primer sequences used in this study were synthesized by Shanghai Sangon Biological Engineering Technology & Services (Shanghai, China) and are listed in Table 1. The qRT-PCR was performed using SYBR Premix Ex Taq™ II Kit on a StepOnePlus™ PCR System (Applied Biosystems, Foster City, CA, USA). The relative expression of target genes was normalized to the expression of *β*-actin, calculated by the 2^−*ΔΔ*CT^ method and given as ratio compared with the control.

### 2.7. Immunofluorescence

EA.hy926 cells were seeded in 6-well plates at a density of 1 × 10^5^ cells per well. After being treated with LiCl and farrerol alone or pretreated with LiCl and then farrerol, the cells were washed with PBS, fixed in 4% paraformaldehyde for 30 min, and permeabilized with 0.5% triton X-100 for 30 min, followed by blocking with 5% BSA for 2 h. Primary anti-Nrf2 monoclonal antibody was added at a dilution of 1 : 200 and allowed to incubate overnight at 4°C. Then, the cells were stained with Alexa Fluor 555-conjugated secondary antibody at room temperature for 1 h and further stained with DAPI for 5 min. Finally, immunofluorescence was observed using a Nikon Eclipse Ti2 inverted fluorescence microscope (Nikon Corporation, Tokyo, Japan) at ×400 magnification. To better visualize the degree of Nrf2 nuclear translocation in digital images, fluorescence intensity surface plots were generated using Nikon NIS Elements Imaging Software (version 4.60). Quantification analysis was performed with the ImageJ software.

### 2.8. Small Interfering RNA (siRNA) Transfection

GSK-3*β* siRNAs, Nrf2 siRNAs, and control siRNAs were designed and synthesized by GenePharma (Shanghai, China). Transient transfection of siRNA was performed with the siRNA-Mate™ transfection reagent according to the manufacturer's instruction. In brief, EA.hy926 cells were cultured in 6-well plates to reach 30%-50% confluency and transfected with GSK-3*β* siRNA, Nrf2 siRNA, or negative control (NC) siRNA for 48 h. Then, the transfected cells were used for further research.

### 2.9. Drug Affinity Responsive Target Stability (DARTS) Assay

DARTS assay was performed as previously reported [25]. Briefly, EA.hy926 cells were lysed with M-PER supplemented with protease and phosphatase inhibitors. After centrifugation (13000 rpm for 15 min at 4°C), an appropriate volume of 10 × TNC (500 mmol/L Tris-HCl pH 8.0, 500 mmol/L NaCl, and 100 mmol/L CaCl_2_) buffer was added into the supernatant and the protein content was determined by the BCA protein assay. All procedures above were performed on ice or at 4°C to prevent premature protein degradation. Lysates were then incubated with various concentrations (160, 320, and 640 *μ*mol/L) of farrerol or DMSO control at room temperature for 30 min. Proteolysis was performed by adding protease (pronase) solution at the ratio of 1 mg pronase to 200 mg of lysate for 30 min at room temperature. The proteolysis was stopped by adding the 5x SDS-PAGE sample loading buffer at 1 : 4 ratio and boiled at 100°C for 10 min. Finally, samples were resolved by SDS-PAGE and the degradation of GSK-3*β* was analyzed by Western blot.

### 2.10. Biolayer Interference (BLI) Assay

The interaction between farrerol and GSK-3*β* was tested by using BLI technology on the ForteBio Octet Red 96e (ForteBio, Inc., American). PBS with 5% DMSO was designated as working buffer throughout the experiment. The recombinant His-tagged GSK-3*β* was immobilized onto Ni-NTA biosensors. Subsequently, the captured biosensors were individually placed into buffer containing various concentrations of farrerol (15.6, 31.3, 62.5, and 125.0 *μ*mol/L) for 60 s (association step) and then transferred into buffer without farrerol for 60 s (dissociation step). The sensors incubated in working buffer without loading protein were used as controls to eliminate background binding and the sensors loading GSK-3*β* without ligand as controls to correct baseline drifts. To determine binding parameters, data were analyzed using ForteBio Data Analysis Software (v.11.1.0.4).

### 2.11. Inhibition of GSK-3*β*

The inhibitory activity of farrerol on GSK-3*β* was evaluated using the Kinase-Glo assay which was performed in assay buffer (50 mmol/L HEPES pH 7.5, 1 mmol/L EDTA, 1 mmol/L EGTA, and 15 mmol/L magnesium acetate) using the black 96-well plate as previously reported [26]. In brief, the reaction system contained a series of different concentrations of the test compound, 20 ng of enzyme, 25 *μ*mol/L substrate, and 1 *μ*mol/L ATP in a total volume of 40 *μ*L. Then, the reaction mixture was incubated at 30°C for 30 min and reaction was stopped by the addition of 40 *μ*L Kinase-Glo reagent. Glow-type luminescence was recorded after 10 min using a SpectraMax i3x microplate reader (Molecular Devices, Sunnyvale, CA, USA). SB 415286, a GSK-3*β* inhibitor, was used as positive control to validate the method in our laboratory. The inhibitory activity was measured indirectly by quantifying the amount of ATP remaining in solution following a kinase reaction. In specific, the luminescent signal is proportional to the amount of ATP remaining in the reaction and the inhibitory activity of the test compound. Maximal kinase (average positive) and luciferase (average negative) activities were used to calculate the inhibitory activity. The former was measured in the absence of inhibitor and the latter in the presence of reference compound inhibitor SB415286 (IC_50_ = 77.5 nmol/L) at total inhibition concentration (5 *μ*mol/L) [27]. The percentage inhibition was calculated by the following equation: inhibition rate (%) = (luminescence of test inhibitor − luminescence of average positive)/(luminescence of average negative − luminescence of average positive) × 100. IC_50_ values were determined by nonlinear regression analysis of GSK-3*β* inhibition versus different concentrations of the test compound using GraphPad Prism 5.0 software (San Diego, CA, USA).

### 2.12. Kinetic Analysis on GSK-3*β*

ADP-Glo kinase assay was used to determine the type of inhibition of farrerol to GSK-3*β*. The kinetic property of GSK-3*β* without or with different concentrations of farrerol (10, 20, and 40 *μ*mol/L) was determined using different concentrations of ATP (0.5, 1, 2, 4, and 8 *μ*mol/L). Kinetic assay was performed in the solid white 96-well plate (80 *μ*L final volume per well) according to the manufacturers' instructions with slight modification. The reaction was carried out for 30 min at 30°C in a total volume of 20 *μ*L and included indicated concentrations of farrerol and ATP, 10 ng of enzyme, and 25 *μ*mol/L substrate. Detection was performed by incubating 20 *μ*L of kinase reaction, 20 *μ*L of ADP-Glo™ Reagent for 40 min, followed by adding 40 *μ*L of Kinase Detection Reagent for 30 min. Luminescence was read using a SpectraMax i3x microplate reader. This assay determined the kinase activity by quantifying the amount of ADP formed in the kinase reaction.

### 2.13. Molecular Docking

The molecular docking software Autodock4.2.6 was employed to illustrate the probable binding mode of farrerol at the active site of GSK-3*β* and find some important residues for binding [28]. Four crystal structures of GSK-3*β* (PDB ID code 4ACC, 4ACD, 4ACG, and 4ACH), determined at resolutions of 2.21 Å, 2.6 Å, 2.6 Å, and 2.6 Å, respectively, were obtained from the Protein Data Bank (https://www.rcsb.org/). For ligand preparation, the structure of farrerol was set up with SYBYL-X 2.0 and finally optimized to minimal energy (tolerance of 0.005 kcal/mol Å) using the Tripos force field with Gasteiger–Hückel charges. Then, the docking input files (PDBQT) for the proteins and ligand were generated by AutoDockTools 1.5.6 [29]. To define the binding site, a cubic grid box of size 60 Å × 60 Å × 60 Å with a spacing of 0.375 Å was generated. The Lamarckian genetic algorithm (LGA) was used to conduct docking simulations and identify appropriate binding modes. Finally, among the obtained conformers in each docking run, the one with the least binding free energy was chosen as the final conformation for further analysis of binding mode. The molecular graphics were generated with AutoDockTools 1.5.6 and Visual Molecular Dynamics 1.9.3 (VMD) [30].

### 2.14. Molecular Dynamics (MD) Simulation

MD simulation was performed using GROMACS (version 2016.4). The conformation with the lowest binding free energy from docking calculations was chosen as the starting structure for MD simulation. The topology and coordinated files of ligand and protein were prepared by antechamber module of AMBER tools 17 using GAFF force field and AMBER99SB-ILDN force field [31] and then converted to GROMACS using the python script acpype.py [32] (https://github.com/t-/acpype). The farrerol-GSK-3*β* complex was immersed in a periodic cubic box (12.96303 × 12.96303 × 12.96303 nm^3^) containing TIP3P water molecules. To electrically neutralize the system, an appropriate number of Cl^−^ ions were added. The minimal distance between the initial complex structure and the edge of the box was set to 12 Å. The final system was composed of the protein and ligand, 68569 water molecules, and 14 negative counterions, for a total of 217075 atoms. The LINCS algorithm was used to constrain bond lengths. The long-range electrostatic interactions were treated by means of the Particle Mesh Ewald (PME) method. A cutoff of 10 Å was used for van der Waals interactions. To relieve unfavorable interactions within the system, energy minimization was conducted using the steepest descent and conjugate gradient methods. Subsequently, a 100 ps equilibration dynamics was performed to remove possible conflicting contacts between solute and solvent. Finally, production simulation was run for 20 ns in the isothermal-isobaric (NPT) ensemble at a temperature of 298.15 K and 1 bar pressure, which were maintained by employing the velocity rescaling and Parrinello-Rahman methods, respectively. During the production simulation, the leapfrog algorithm with a time step of 2 fs was used to integrate the equations of motion and the trajectory was recorded every 2 ps. Furthermore, the binding free energy of GSK-3*β* with farrerol was calculated using g_mmpbsa tool with default settings [33, 34].

### 2.15. Statistical Analysis

All results are expressed as means ± standard deviation (SD). To assess the statistical significance, Student's *t*-test for comparisons between two groups was conducted. *p* values less than 0.05 were considered to indicate statistical significance.

## 3. Results

### 3.1. Cytotoxicity of Farrerol on EA.hy926 Cells

First, the cytotoxicity of farrerol in the EA.hy926 cells was investigated with the MTT assay. As illustrated in Figure 1(b), no cellular toxicity was observed at concentrations of up to 80 *μ*mol/L for 24 h. Therefore, in this study, farrerol was used at a concentration between 10 and 80 *μ*mol/L in subsequent experiments.

### 3.2. Effects of Farrerol on the Expression of Nrf2-Associated Antioxidant Enzymes and Nrf2 Nuclear Translocation

**Figure 2:**
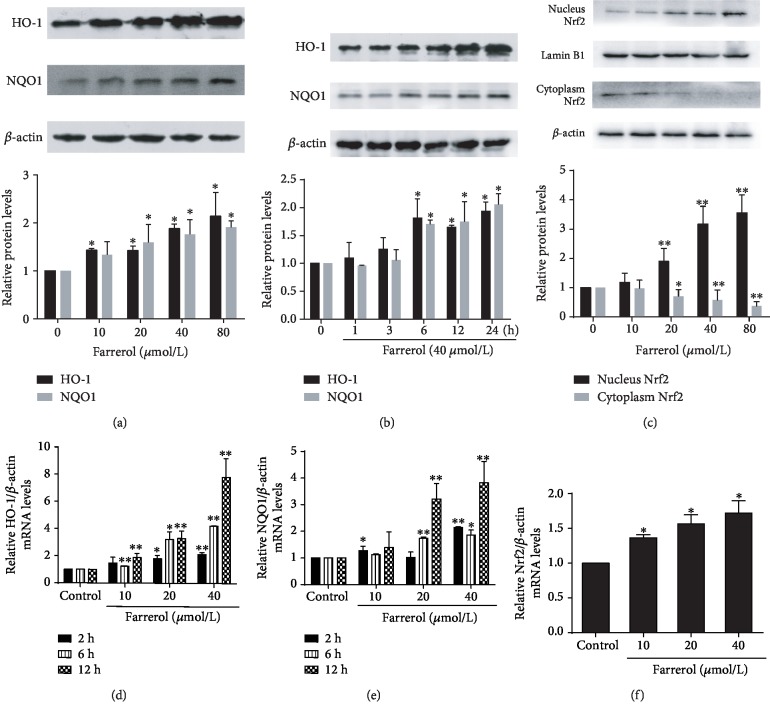
Effects of farrerol on Nrf2-associated antioxidant enzyme expression and Nrf2 nuclear translocation in EA.hy926 cells. (a, b) The protein expression levels of HO-1 and NQO1 were measured by Western blot assay. The *β*-actin protein level was considered as an internal control. (c) The nuclear extracts, cytosolic extracts, and total cell lysates were prepared, and the protein expression of Nrf2 was examined by Western blot. Lamin B1 and *β*-actin were used as loading controls for nuclear extracts and cytosolic extracts, respectively. (d–f) Total RNA was extracted from EA.hy926 cells treated as indicated. The mRNA expression of HO-1, NQO1, and Nrf2 was determined by real-time PCR. The values shown represent the means ± SD obtained for three independent experiments. ^∗^*p* < 0.05 and ^∗∗^*p* < 0.01 compared to the control group.

HO-1 and NQO1 are considered as representative antioxidant enzymes that play crucial roles in the cellular defense system against oxidative stress. Since farrerol could markedly attenuate H_2_O_2_-induced intracellular ROS production in EA.hy926 cells [19], the ability of farrerol to stimulate the protein expression of HO-1 and NQO1 was examined by Western blot analysis. As shown in Figures 2(a) and 2(b), the treatment of EA.hy926 cells with farrerol at concentrations of 10, 20, 40, and 80 *μ*mol/L for 24 h significantly enhanced HO-1 and NQO1 protein expression in a dose-dependent manner, and the treatment of cells with 40 *μ*mol/L farrerol resulted in a time-dependent increase in HO-1 and NQO1 protein expression.

As both HO-1 and NQO1 are typical downstream antioxidant enzymes mediated by Nrf2, the effects of farrerol on Nrf2 activation and mRNA expression of HO-1 and NQO1 were further assessed. Results in Figures 2(c) and 2(f) showed that farrerol facilitated its nuclear accumulation and increased the mRNA expression of Nrf2 in a dose-dependent manner. Also, farrerol dose- and time-dependently induced HO-1 and NQO1 mRNA expression (Figures 2(d) and 2(e)). These results suggest that farrerol activates the Nrf2-ARE pathway in EA.hy926 cells.

### 3.3. Nrf2 siRNA Attenuated Farrerol-Mediated Cytoprotective Effect and Induction of HO-1 and NQO1

**Figure 3:**
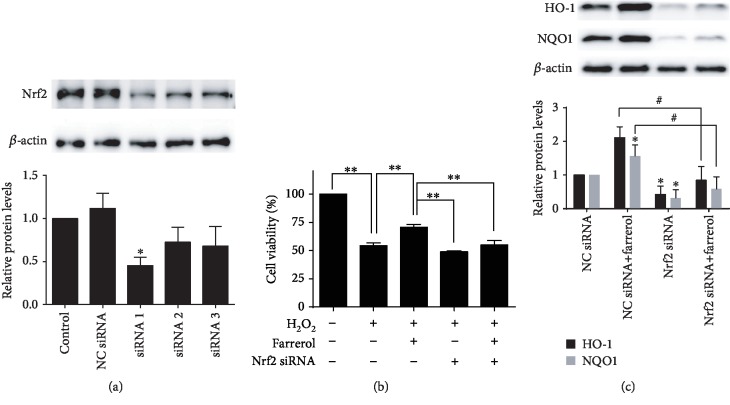
Nrf2 silencing attenuated farrerol-mediated cytoprotective effect and induction of HO-1 and NQO1. (a) Nrf2 knockdown efficiency at protein level was detected by Western blot. Data are presented as means ± SD (*n* = 3). ^∗^*p* < 0.05 compared to the control group. (b) Nrf2 silencing reduced the cytoprotective effects of 40 *μ*mol/L farrerol on H_2_O_2_-induced cell damage. Data are presented as means ± SD (*n* = 3). ^∗∗^*p* < 0.01 compared to the indicated group. (c) The protein expression of HO-1 and NQO1 was examined by Western blot. Data are presented as means ± SD (*n* = 3). ^#^*p* < 0.05 compared to the indicated group, ^∗^*p* < 0.05 compared to the NC siRNA-treated group.

To further verify the role of Nrf2 in the regulation of antioxidant protein expression by farrerol, three siRNAs targeting Nrf2 were constructed and the most effective siRNA (siRNA 1) was selected for the subsequent experiments (Figure 3(a)). The effect of Nrf2 silencing on farrerol-mediated endothelial protective effect against oxidative damage was detected. After being transfected with Nrf2 siRNA or NC siRNA, the cells were incubated with 40 *μ*mol/L farrerol for 24 h and then stimulated with or without 200 *μ*mol/L H_2_O_2_ for a further 6 h. The results from MTT assay revealed that the cytoprotective effects of farrerol were significantly reduced by siRNA-induced knockdown of Nrf2 (Figure 3(b)). Then, Western blot was employed to further examine the impact of Nrf2 silencing on farrerol-mediated induction of HO-1 and NQO1. As shown in Figure 3(c), Nrf2 silencing significantly suppressed farrerol-induced upregulation of HO-1 and NQO1. These results demonstrate that farrerol-induced cytoprotective effect and expression of HO-1 and NQO1 are mediated by the activation of Nrf2 in EA.hy926 cells.

### 3.4. Farrerol Induced Akt/GSK-3*β* Phosphorylation in EA.hy926 Cells

**Figure 4:**
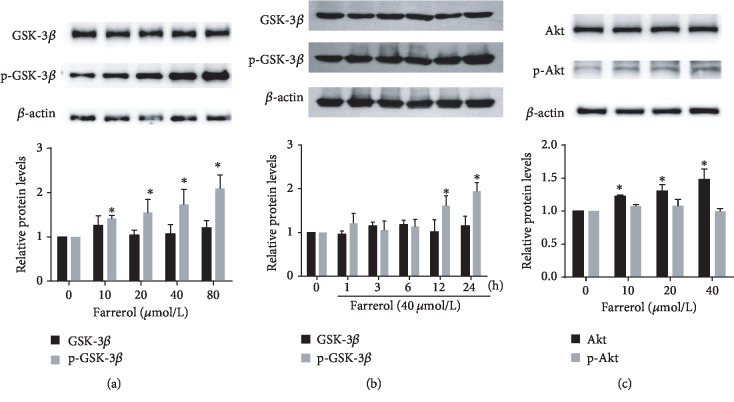
Effects of farrerol on GSK-3*β* phosphorylation in EA.hy926 cells. (a, b) Farrerol induced GSK-3*β* phosphorylation at Ser9 without affecting GSK-3*β* expression in a dose- and time-dependent manner. (c) Farrerol increased the phosphorylation level of Akt without significantly altering the levels of total Akt in a dose-dependent manner. The data in the figures represent the means ± SD (*n* = 3). ^∗^*p* < 0.05 compared to the control group.

It is previously reported that Nrf2 is regulated by GSK-3*β* in a Keap1-independent way and some phytochemicals including flavonoids activate Nrf2 through inhibitory phosphorylation of GSK-3*β* [35–37]. To explore this possibility, the impact of farrerol treatment on phosphorylation of GSK-3*β* was monitored. As illustrated in Figures 4(a) and 4(b), farrerol dose- and time-dependently enhanced the phosphorylation level of GSK-3*β* at Ser9 without affecting the protein expression level of GSK-3*β*. Additionally, Akt, an upstream molecule of GSK-3*β*/Nrf2 signaling, was also examined. As shown in Figure 4(c), treatment with farrerol dose-dependently increased the phosphorylation level of Akt without significantly altering the levels of total Akt. These findings imply that the activation of the Nrf2-ARE signaling pathway by farrerol depends, at least partially, on the inactivation of GSK-3*β*.

### 3.5. LiCl, a GSK-3*β* Inhibitor, Increased Farrerol-Mediated Cytoprotective Effect and Nrf2 Nuclear Accumulation

**Figure 5:**
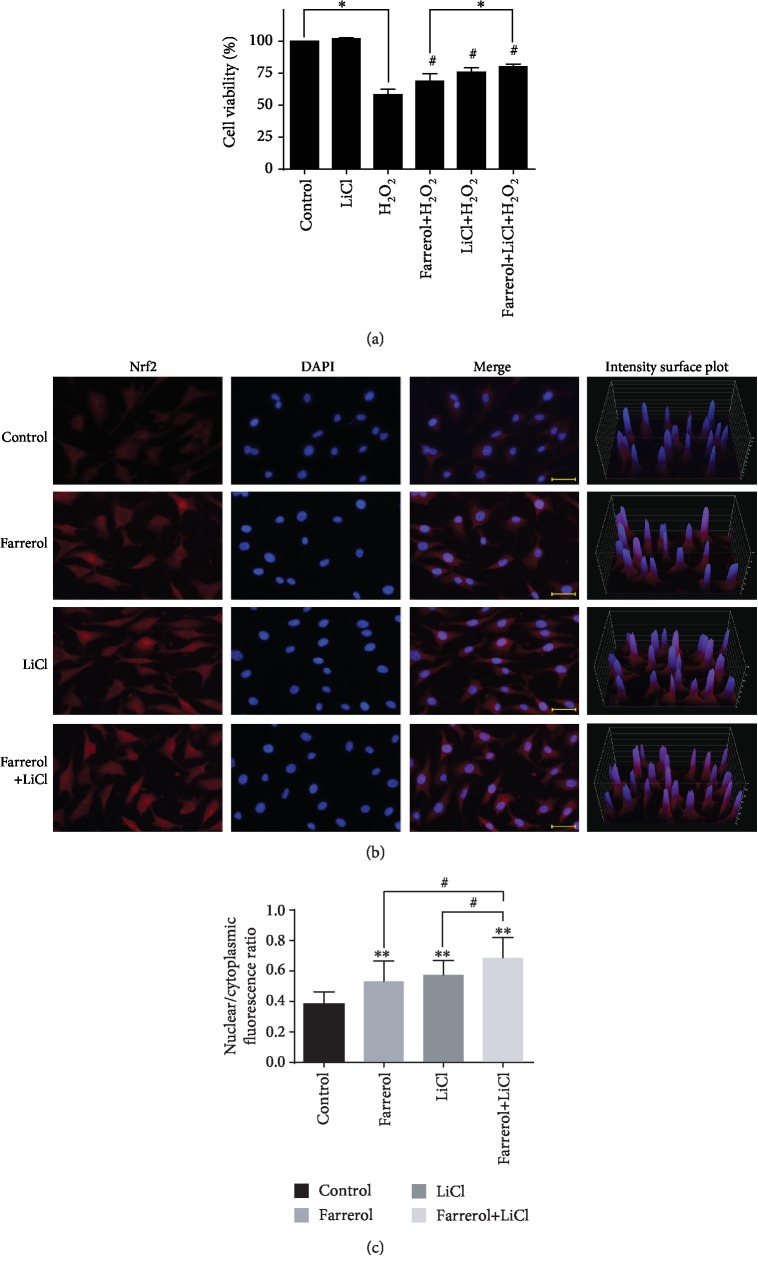
GSK-3*β* inhibitor LiCl enhances farrerol-mediated cytoprotective effect and Nrf2 nuclear accumulation in EA.hy926 cells. (a) LiCl enhanced the cytoprotective effects of 40 *μ*mol/L farrerol on H_2_O_2_-induced cell damage. ^∗^*p* < 0.05 compared to the indicated group. ^#^*p* < 0.05 compared to the H_2_O_2_-treated group. (b) The nuclear translocation of Nrf2 was detected by immunofluorescence combined with DAPI staining for nuclei. Scale bar = 50 *μ*m. (c) The nuclear translocation of Nrf2 was assessed as a ratio of nuclear to cytoplasmic fluorescence using the ImageJ software (*n* = 10). ^∗∗^*p* < 0.01 compared to the control group. ^#^*p* < 0.05 compared to the indicated group.

LiCl, a specific inhibitor of GSK-3*β*, was used to further explore the role of GSK-3*β* in the farrerol-mediated protection against H_2_O_2_-induced oxidative damage in EA.hy926 cells. After being preincubated with 10 mmol/L LiCl for 30 min, the cells were incubated with 40 *μ*mol/L farrerol for 24 h and then stimulated with or without 200 *μ*mol/L H_2_O_2_ for a further 6 h. The results from MTT assay demonstrated that the cytoprotective effects of farrerol were enhanced by the addition of LiCl (Figure 5(a)). In addition, immunofluorescence analysis revealed that nuclear Nrf2 protein levels in the cells treated with farrerol and LiCl alone were significantly increased when compared to those in vehicle control cells Figures 5(b) and 5(c). Moreover, enhanced nuclear Nrf2 protein levels were observed in the cells treated with farrerol and LiCl in combination compared with individual effects of each agent alone. These results demonstrate that the inactivation of GSK-3*β* participates in the action of farrerol against H_2_O_2_-induced oxidative damage in EA.hy926 cells.

### 3.6. siRNA-Mediated Inhibition of GSK-3*β* Enhanced Farrerol-Induced Activation of the Nrf2-ARE Pathway

**Figure 6:**
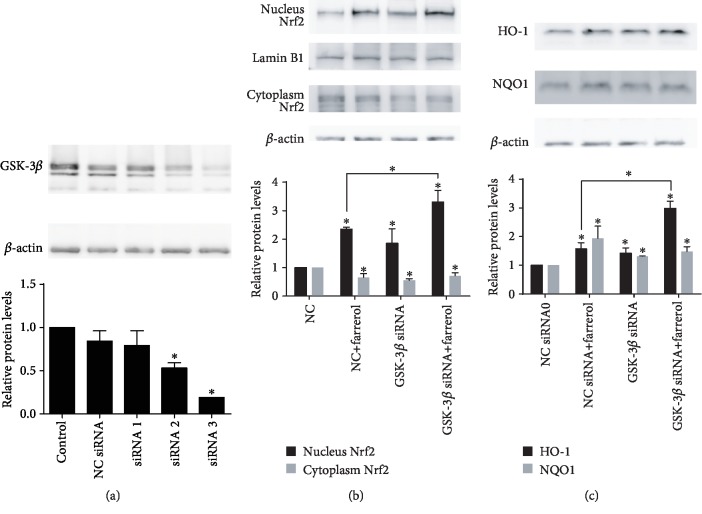
Effects of GSK-3*β* siRNA-mediated GSK-3*β* inhibition on farrerol-induced activation of the Nrf2-ARE pathway in EA.hy926 cells. (a) GSK-3*β* knockdown efficiency at protein level was detected by Western blot. (b) Cytoplasmic and nuclear levels of Nrf2 were detected by Western blotting to analyze the translocation of Nrf2. Lamin B1 and *β*-actin were used as loading controls for nuclear and cytosolic protein fractions, respectively. (c) The protein expression of HO-1 and NQO1 was examined by Western blot. Data are presented as means ± SD (*n* = 3); ^∗^*p* < 0.05.

To further elucidate the role of GSK-3*β* inhibition in farrerol-induced Nrf2-ARE pathway activation, three siRNAs targeting GSK-3*β* were constructed and the most effective siRNA (siRNA 3) was selected and used (Figure 6(a)). As shown in Figure 6(b), GSK-3*β* silencing significantly promoted Nrf2 nuclear translocation and upregulated HO-1 and NQO1 protein expression. Moreover, GSK-3*β* silencing also facilitated farrerol-induced nuclear accumulation of Nrf2 and upregulation of HO-1 protein expression, but not the upregulation of NQO1 protein expression (Figure 6(c)). These results indicate that GSK-3*β* is involved in farrerol-mediated Nrf2-ARE signaling pathway activation in EA.hy926 cells.

### 3.7. Farrerol Directly Bound to GSK-3*β*

**Figure 7:**
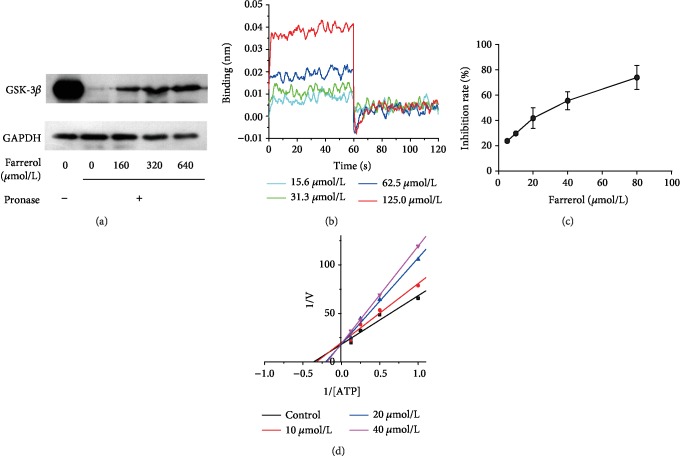
GSK-3*β* is a direct target protein for farrerol. (a) Farrerol protects GSK-3*β* against proteolysis in DARTS assays. (b) BLI analysis of farrerol binding to GSK-3*β*. (c) The kinase activity of GSK-3*β* in different concentrations of farrerol. (d) Lineweaver-Burk plots. ATP concentration varied from 1 to 8 *μ*mol/L; phospho-glycogen synthase peptide-2 concentration was kept constant at 25 *μ*mol/L; farrerol concentrations were depicted in the plot.

To detect whether farrerol could directly interact with GSK-3*β*, DARTS assay was applied. DARTS assay is an effective method to identify protein-small molecule interactions and developed based on the principle that proteolysis resistance of the target protein is enhanced by the interaction with small molecular ligand. As depicted in Figure 7(a), an increase in abundance of GSK-3*β* band was observed with increasing dose of farrerol. Meanwhile, GAPDH was used as loading control owing to its stability in the same conditions. To further confirm the interaction between farrerol and GSK-3*β*, BLI assay was performed for binding affinity measurement. As shown in Figure 7(b), farrerol could bind to GSK-3*β* in a concentration-dependent manner, with an equilibrium dissociation constant (*K*_*D*_) value of 6.338 × 10^−4^ mol/L. Thus, these data suggest that there exists a direct and specific binding between GSK-3*β* and farrerol.

### 3.8. Inhibitory Effect of Farrerol on GSK-3*β In Vitro*

To investigate whether farrerol could regulate the protein kinase activity of GSK-3*β*, the optimized Kinase-Glo assay was employed [26]. As shown in Figure 7(c), farrerol exhibited expected inhibition on GSK-3*β* in a dose-dependent manner. The concentration of farrerol leading to a loss of 50% enzyme activity (IC_50_) was determined to be 27.14 ± 1.08 *μ*mol/L. Also, to verify the method in our laboratory conditions and estimate the accuracy, SB415286, a specific GSK-3*β* inhibitor, was used as positive control and its IC_50_ value was obtained to be 48.79 ± 1.04 nmol/L, which was consistent with the literature value (77.5 nmol/L) [27]. These results indicate that farrerol is a direct GSK-3*β* inhibitor *in vitro*.

### 3.9. Kinetic Mode of Inhibition of Farrerol on GSK-3*β*

To clarify the inhibition mechanism of farrerol on GSK-3*β*, Lineweaver-Burk analyses were performed. Briefly, the kinetic analyses were conducted using combinations of four inhibitor concentrations from 0 to 40 *μ*mol/L and five ATP concentrations. The Lineweaver-Burk double reciprocal plots were generated by linear least-square fits of the data. As depicted in Figure 7(d), all the generated lines showed a coinciding intercept on the vertical axis, indicating an ATP-competitive inhibition mechanism. These kinetic data suggest that farrerol is an ATP-competitive inhibitor of GSK-3*β*.

### 3.10. Molecular Docking of Farrerol Binding to GSK-3*β*

**Figure 8:**
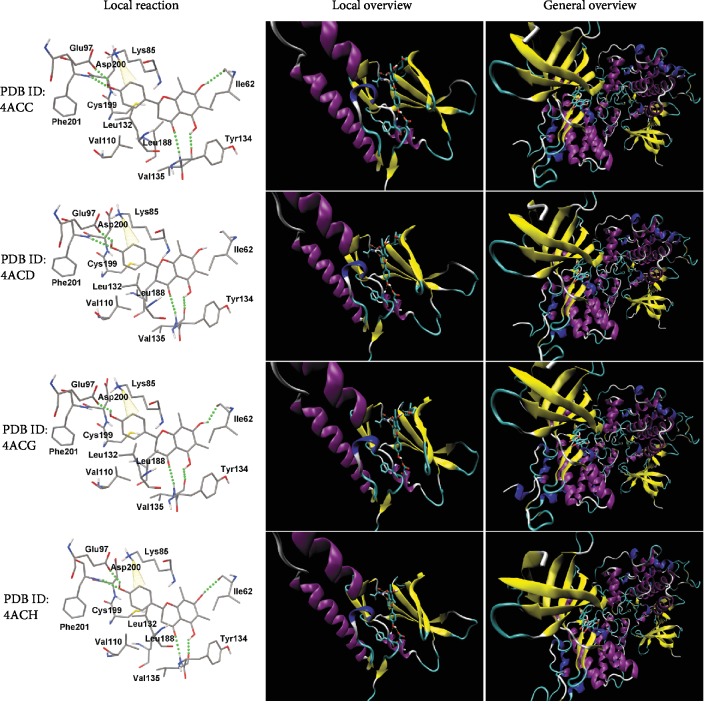
Docking binding mode of farrerol in the ATP-binding site of GSK-3*β*.

To further elucidate the binding mode between farrerol and GSK-3*β* at the molecular level, molecular docking simulation was carried out. Since the experimental results suggest an ATP-competitive inhibition mechanism against GSK-3*β*, farrerol was docked into the ATP binding site of GSK-3*β*. The 4 selected PDB structures without missing residues in the middle of the chain were used. As presented in Figure 8, the best-scoring conformations of farrerol obtained from each docking run showed the same orientation and a similar disposition. Farrerol deeply penetrated into the binding pocket with 4′-hydroxyl of the ring B directed towards the depths of the cavity and was surrounded by the amino acid residues, such as Ile62, Lys85, Glu97, Val110, Leu132, Tyr134, Val135, Leu188, Cys199, Asp200, and Phe201. Molecular interactions between farrerol and GSK-3*β* consisted of hydrogen bond and pi-cation interaction. Although the number of formed hydrogen bond varied (3 to 4), the free binding energy was found to be almost identical for each of the four conformations (interaction details shown in Table 2). These docking studies propose reasonable binding models of the farrerol-GSK-3*β* complex and provide an in-depth understanding of the interactions between farrerol and GSK-3*β*.

### 3.11. Molecular Dynamics Simulation Analysis

In order to further evaluate the structural stability of the farrerol-GSK-3*β* complex and the interactions between farrerol and GSK-3*β* simultaneously considering the effects of solvent, temperature, and pressure as well as the flexibility of protein, MD simulation was performed in NPT ensemble.

**Figure 9:**
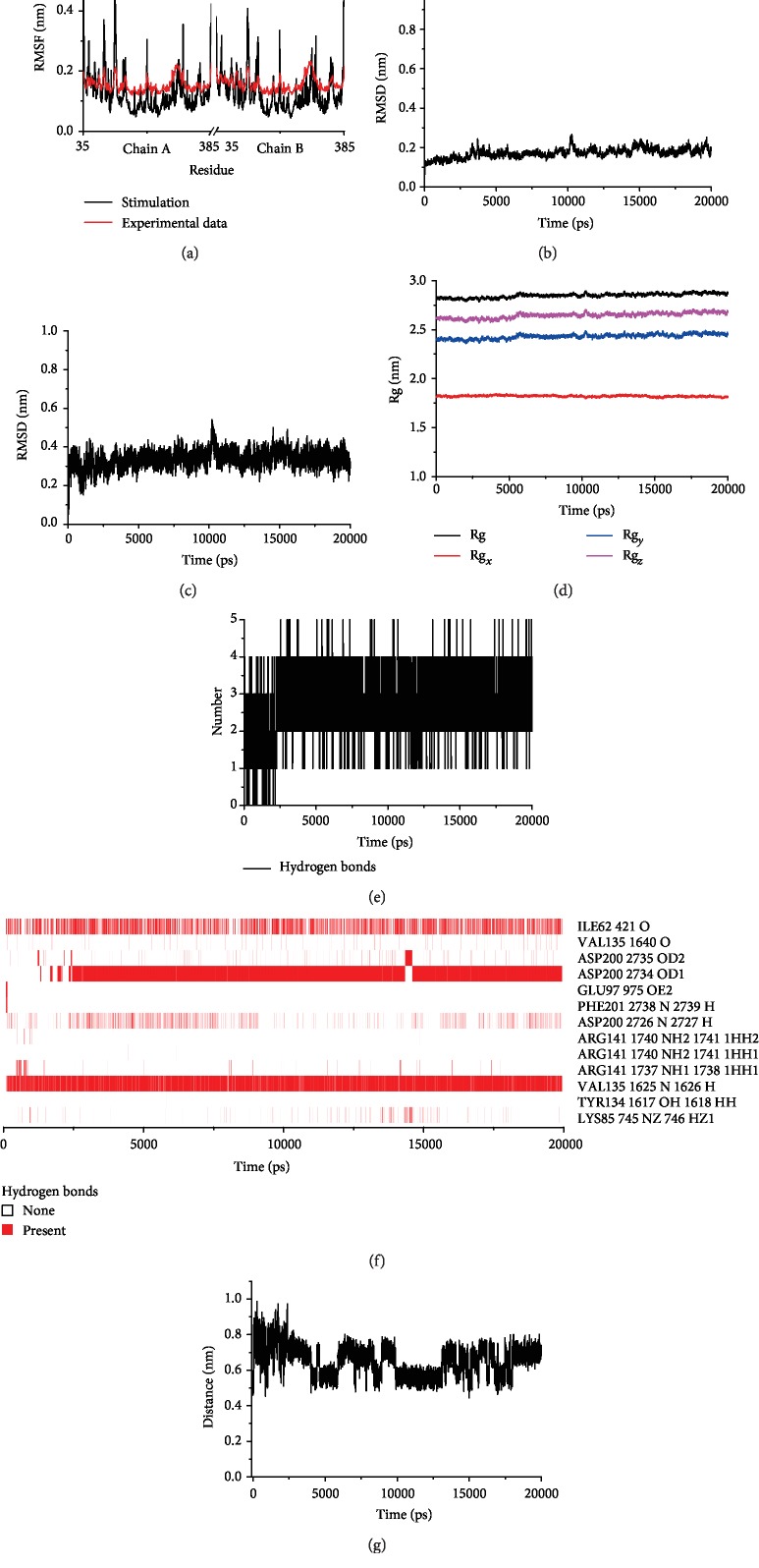
MD simulation trajectory analysis. (a) RMSF of native structure of GSK-3*β* (complex with the cocrystal inhibitor GR9) as well as GSK-3*β* in complex with farrerol. (b, c) RMSD of protein backbone and farrerol obtained during the 20 ns MD simulation. (d) Rg of the GSK-3*β*-farrerol complex obtained during 20 ns of MD simulation. (e) Time evolution plot of the intermolecular hydrogen bond numbers between GSK-3*β* and farrerol. (f) Hydrogen bond existence map for the GSK-3*β*-farrerol complex during the 20 ns MD simulation. (g) The distance between the H (HZ2) atom of Lys85 and the center of the benzene group (ring B of farrerol) in the 20 ns simulations.

To assess whether the simulation results are reasonable and reliable, the root mean square fluctuation (RMSF) values of GSK-3*β* in complex with farrerol and the native GSK-3*β* (in complex with a synthesized inhibitor) were obtained and then compared with each other. It should be noted that RMSF values reflect the mobility of the individual residues and those of GSK-3*β* in the two complexes described above were obtained in two different ways. In specific, RMSF values over the simulated trajectory were analyzed utilizing the gmx rmsf tool included in the GROMACS package, while crystallographic RMSF values for each residue were converted from B factor values in the PDB file using ba2r program (http://sobereva.com/32). Then, the RMSF values obtained from the trajectory data were compared to the converted ones. As shown in Figure 9(a), the trend of the curve (especially in the region where the residues were not located in the binding pocket) obtained from MD simulation was basically consistent with the result calculated based on experimental data, which is an indication of the rationality of the simulation conditions.

In addition, the root mean square deviation (RMSD) and the radius of gyration (Rg) were analyzed to examine the dynamic stability of the farrerol-GSK-3*β* complex. RMSD is a parameter defined to quantify the structural change in the protein and ligand structures during MD simulation. As illustrated in Figures 9(b) and 9(c), the protein backbone and ligands' RMSD was plotted as a function of time. During the simulation, calculated average RMSD values of the protein backbone and ligand were found to be 0.17 ± 0.02 and 0.34 ± 0.05 nm, respectively, indicating the structure of the farrerol-GSK-3*β* complex reached a stable and equilibrium state in water after small adjustments and the structural stability was maintained to the end of simulation. Rg is an effective tool to measure the structural integrity and compactness of overall protein at different time points and can be defined as the root mean square distance of the collection of atoms from their common center of gravity. A time evolution plot of Rg (Figure 9(d)) showed that GSK-3*β* in complex with farrerol had stable trajectory throughout the entire simulation. This result further suggests the structure stability of the farrerol-GSK-3*β* complex.

Furthermore, to better understand the interactions between farrerol and GSK-3*β*, hydrogen bond and pi-cation interactions were analyzed on the basis of MD trajectory. The inspection of time evolution plot of the intermolecular hydrogen bond numbers (Figure 9(e)) showed that a maximum of 5 hydrogen bonds was present in the complex between farrerol and GSK-3*β*, and on average, the number of hydrogen bonds was between 2 and 3. Moreover, the hydrogen bond existence map for the farrerol-GSK-3*β* complex was represented in Figure 9(f) and showed that farrerol formed stable hydrogen bonds with Val135 and Asp200 of the active site. The hydrogen bond formed with Val135 was maintained throughout the entire simulation time, while the one formed with Asp200 occurred after 2.5 ns. Also, it could be observed that an unstable hydrogen bond between farrerol and Ile62 appeared periodically. Additionally, considering the docking results, pi-cation interaction between farrerol and Lys85 was examined and the distance between the H (HZ2) atom of Lys85 and the center of the benzene group (ring B of farrerol) was calculated. As illustrated in Figure 9(g), the distance increased quickly in initial 60 ps and then oscillated between 0.4 and 1.0 nm, indicating pi-cation interaction was weak and even disappeared during the simulation. The analysis results suggest that hydrogen bonds contribute substantially to the stability of the farrerol-GSK-3*β* complex.

To provide a deeper insight into the molecular interactions between farrerol and GSK-3*β*, the binding free energy and its corresponding components, such as van der Waals energy, electrostatic energy, polar solvation energy, and SASA energy, were calculated using MM-PBSA method implemented in g_mmpbsa tool. As listed in Table 3, the farrerol-GSK-3*β* complex possessed a negative binding free energy of −65.236 ± 10.563 kJ/mol. Among the four components, van der Waals, electrostatic, and SASA energy contributed negatively to the total binding free energy, whereas polar solvation energy contributed positively. Both van der Waals and electrostatic energy components played a key role in the binding of farrerol and GSK-3*β*, and the contribution of van der Waals energy component was almost three times greater than that of the electrostatic energy component. These results indicate that farrerol could form a stable binding with GSK-3*β* and the intermolecular binding interaction is a spontaneous process.

## 4. Discussion

Pathological studies have shown that endothelial dysfunction is the initial step in the pathogenesis of atherosclerosis and is associated with most forms of cardiovascular disease [2, 3]. Moreover, it has been acknowledged that oxidative stress is one of the critical risk factors for endothelial dysfunction [4]. Therefore, reduction of endothelial oxidative stress might be beneficial in reducing the risk of cardiovascular diseases. In the past decades, flavonoids attract much attention in the prevention of endothelial dysfunction and its related cardiovascular diseases. Extensive studies indicate that flavonoids have a positive influence on various endothelial dysfunction-related cardiovascular diseases and the effects are attributed to their antioxidant activity [6, 7].

Farrerol, a naturally occurring flavanone extracted from the leaves of edible and medicinal plant *Rhododendron dauricum* L., has been proved to exert protective effects against H_2_O_2_-induced damage in human endothelium cells and positive influence on cardiovascular diseases in our previous study. We showed that farrerol prevented H_2_O_2_-induced endothelial tight junction disruption and endothelial cell apoptosis, which were likely associated with modulation of ERK1/2 and p38 activation, respectively. In addition, cellular experiments revealed that farrerol restored the activities of superoxide dismutase (SOD) and glutathione peroxidase (GSH-Px) and reduced the intracellular malondialdehyde (MDA) and ROS levels in H_2_O_2_-challenged endothelial cells [19, 20]. However, the possible underlying antioxidant cellular mechanisms and targets that are involved in the protection effects remain to be illustrated.

In the present study, we demonstrated that the Nrf2-ARE pathway was activated by farrerol in EA.hy926 cells. The Nrf2-ARE pathway plays a significant role in response to oxidative stress by triggering the production of corresponding antioxidant enzymes and phase II detoxifying enzymes [38]. We found that farrerol was capable of inducing HO-1 and NQO1 expression in protein and mRNA level and promoting Nrf2 nuclear translocation. Moreover, Nrf2 siRNA attenuated farrerol-mediated cytoprotective effect and induction of HO-1 and NQO1. These results are consistent with previous findings that farrerol attenuates oxidative stress through activating the Nrf2-ARE pathway in BV-2 cells and RAW 264.7 cells [14, 24]. Interestingly, we also found that farrerol enhanced the mRNA expression of Nrf2, which could not be explained and awaits further study.

Recent studies indicate that apart from Keap1, the Nrf2-ARE pathway is also modulated by GSK-3*β* [23]. GSK-3*β* is tightly regulated by a variety of upstream protein kinases represented by PI3K/Akt, ERK1/2, and p38 [39–41]. As our previous study revealed that modulation of ERK1/2 and p38 activation participate in farrerol-mediated endothelial cell protection against oxidative stress, we hypothesized whether GSK-3*β* was involved in the farrerol-induced Nrf2-ARE pathway activation in vascular endothelial cells. Therefore, the effect of farrerol on the expression and phosphorylation of GSK-3*β* was detected at first. Results showed that the phosphorylation of GSK-3*β* at Ser9 was induced by farrerol in a dose- and time-dependent manner, while the protein levels of GSK-3*β* were not significantly influenced. In addition, we examined the activation of Akt in EA.hy926 cells treated with farrerol. No change in the expression of total Akt was detected, whereas Akt phosphorylation was significantly increased in a dose-dependent manner. These results are consistent with previous findings which indicate that farrerol increased Akt phosphorylation in a dose-dependent manner in HepG2 cells and RAW 264.7 cells [14, 42]. Taking these and our previous results into account, GSK-3*β* might act as a major integrator of multiple signaling (e.g., PI3K/Akt, ERK1/2, and p38). Then, the GSK-3*β* inhibitor LiCl and GSK-3*β* siRNA were further used to verify the role of GSK-3*β* in Nrf2-ARE pathway activation. Our findings indicated that LiCl enhanced the cytoprotective effects of farrerol and facilitated Nrf2 translocation into nuclear. Moreover, siRNA-mediated inhibition of GSK-3*β* effectively promoted Nrf2 nuclear translocation and upregulated HO-1 protein expression. These results are in accord with recent studies indicating that the flavonoids puerarin, butin, and hyperoside induced an increase in phosphorylation of GSK-3*β* at Ser9 without affecting the protein expression of GSK-3*β* and enhanced Nrf2 nuclear translocation in PC12 cells, H9c2 cells, and L02 cells, respectively [36, 40, 43]. This confirms that GSK-3*β* participates in farrerol-mediated endothelial cytoprotective effects and upregulation of the Nrf2-ARE signaling pathway.

The expression of both HO-1 and NQO1 could be induced by farrerol in EA.hy926 cells which is in agreement with a recent study showing that farrerol could induce the expression of HO-1 and NQO1 in RAW 264.7 cells [14]. Interestingly, siRNA-mediated inhibition of GSK-3*β* promoted farrerol-induced upregulation of HO-1, but failed to facilitate farrerol-induced upregulation of NQO1. The following two points should be considered regarding the observed results. Firstly, GSK-3*β* siRNA transfection may affect Nrf2 binding to specific subsets of ARE sites and finally the selective activation of the ARE-regulated genes. Secondly, considering the complexity of the Nrf2-ARE pathway and its diverse regulatory mechanisms, there is a possibility that unknown factors should be involved. Kannan et al. reported that GSK3 mediated the FBW7- (F-box/WD Repeat-containing Protein 7-) dependent protein degradation of NFE2L3 (Nuclear Factor, Erythroid 2-Like 3) transcription factor, which significantly repressed NQO1 ARE-driven luciferase activity [44]. Therefore, we speculate that NFE2L3 or other unknown factors may lead to the observed results. Obviously, this possibility needs to be verified in future work.

To further explore the role of GSK-3*β* in the farrerol-exerted protective effects against oxidative stress-induced endothelial injuries, we explored whether there exists physical interaction between farrerol and GSK-3*β*. First, DARTS and BLI assays were employed to detect the binding affinity. The results suggested that farrerol could specifically bind to GSK-3*β*. Subsequently, *in vitro* kinase experiment revealed that farrerol inhibited the kinase activity of GSK-3*β* with an IC_50_ of 27.14 ± 1.08 *μ*mol/L. Further, Lineweaver-Burk analysis of the binding interaction demonstrated that farrerol behaved as an ATP-competitive inhibitor. These findings are in agreement with a recent X-ray diffraction study showing that morin, a natural flavonol, could inhibit GSK-3*β* by binding to the ATP binding pocket [45]. Thereafter, to further clarify the binding mode between farrerol and GSK-3*β* at the molecular level, molecular docking and molecular dynamics simulation were carried out. The farrerol activity site (ATP binding site of GSK-3*β*) interaction was predicted by molecular docking, and the best docking pose was further evaluated using molecular dynamics simulations. Our study revealed that farrerol formed stable hydrogen bonds with Val135 and Asp200 of the active site, which were reported to be the key residues in the binding cavity [46]. Moreover, the negative binding free energy of the farrerol-GSK-3*β* complex confirmed a favorable interaction between farrerol and GSK-3*β*, which are consistent with our experimental results. The data presented here confirm the hypothesis that farrerol could inhibit the kinase activity of GSK-3*β* through direct and specific interaction, not just by inducing the inhibitory phosphorylation.

**Figure 10:**
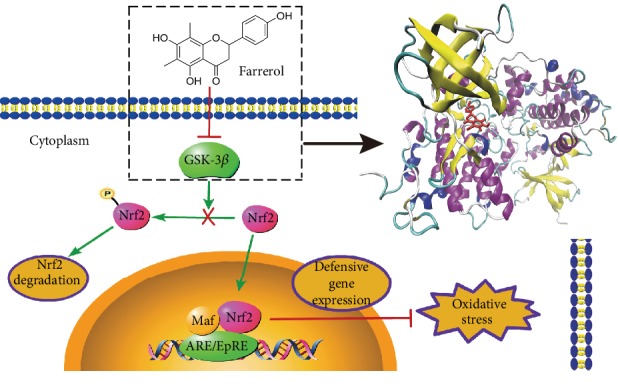
Schematic presentation of probable protective mechanism of farrerol against oxidative stress injury in EA.hy926 cells. Farrerol directly inhibited the kinase activity of GSK-3*β* to activate the Nrf2-ARE pathway and further attenuate oxidative stress damage in EA.hy926 cells.

In summary, we found for the first time that farrerol attenuates H_2_O_2_-induced oxidative damage in EA.hy926 cells through GSK-3*β*-mediated Nrf2-ARE signaling pathway activation. Most notably, we provided evidence that farrerol could not only induce the inhibitory phosphorylation of GSK-3*β* but also directly target GSK-3*β* to inhibit its kinase activity. These observations indicate that GSK-3*β* is a potential target for farrerol to attenuate oxidative stress-induced endothelial damage (Figure 10). Collectively, our findings provide a new target for further structural optimization of flavanones which have the same core structure as farrerol in the treatment of oxidative stress-associated cardiovascular diseases.

## Figures and Tables

**Table 1:** Primer sequences used for qRT-PCR.

Gene	Forward primer	Reverse primer
HO-1	AAGACTGCGTTCCTGCTCAAC	AAAGCCCTACAGCAACTGTCG
NQO1	GAAGAGCACTGATCGTACTGGC	GGATACTGAAAGTTCGCAGGG
Nrf2	TCCAGTCAGAAACCAGTGGAT	GAATGTCTGCGCCAAAAGCTG
*β*-actin	CATGTACGTTGCTATCCAGGC	CTCCTTAATGTCACGCACGAT

**Table 2:** Molecular interactions between farrerol and GSK-3*β*.

GSK-3*β* PDB ID code	Binding energy (kcal mol^−1^)	Hydrogen bond formation				Pi-cation interaction	
		Number	Residues involved in hydrogen bond formation	Atoms of farrerol involved in hydrogen bond formation	Hydrogen bond length (Å)	Residues involved in pi-cation interaction	Distance*^a^* (Å)
4ACC	-8.57	5	Ile62	7-OH	3.14	Lys85	4.00
			Glu97	4′-OH	2.55		
			Val135	5-OH	2.63		
			Val135	4-C=O	3.18		
			Phe201	4′-OH	3.13		

4ACD	-8.58	4	Glu97	4′-OH	2.56	Lys85	4.34
			Val135	5-OH	2.50		
			Val135	4-C=O	3.01		
			Phe201	4′-OH	3.11		

4ACG	-8.55	4	Ile62	7-OH	2.97	Lys85	4.19
			Glu97	4′-OH	2.48		
			Val135	5-OH	2.59		
			Val135	4-C=O	3.04		

4ACH	-8.49	5	Ile62	7-OH	3.04	Lys85	4.35
			Glu97	4′-OH	2.55		
			Val135	5-OH	2.59		
			Val135	4-C=O	2.98		
			Phe201	4′-OH	3.10		

^a^Data are expressed as the distance between the H (HZ2) atom of Lys85 and the center of the benzene ring (ring B).

**Table 3:** Binding energy and individual component energy values for the farrerol-GSK-3*β* complex.

Energetic terms (kJ/mol)	
van der Waals energy	−140.970 ± 11.106
Electrostatic energy	−47.991 ± 15.612
Polar solvation energy	139.272 ± 13.209
SASA energy*^a^*	−15.547 ± 0.685
Binding energy	−65.236 ± 10.563

^a^SASA: solvent accessible surface area.

## Data Availability

The data used to support the findings of this study are included within the article.
